# Supplementary material for: Adaptive radiation by waves of gene transfer leads to fine-scale resource partitioning in marine microbes
Source: Nat Commun. 2016 Sep 22;7:12860. doi: 10.1038/ncomms12860 (PMC5036157; doi:10.1038/ncomms12860)
Supplement: Supplementary Information — Supplementary Figure 1-10, Supplementary Tables 1-3 and Supplementary References. [file ncomms12860-s1.pdf]

Supplementary Figures

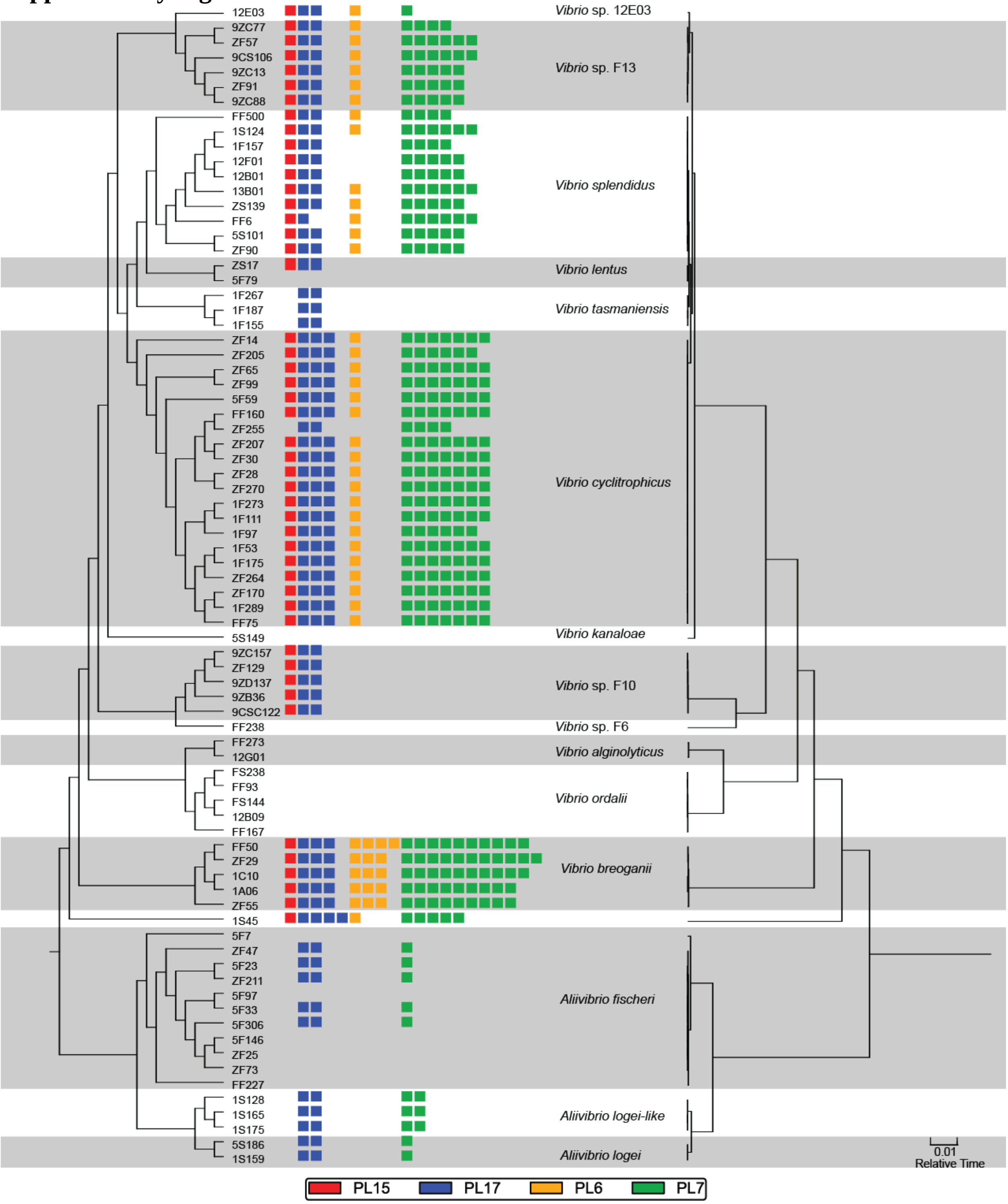

Supplementary Figure 1. Aly and Oal domain dosage with maximum likelihood phylogeny of 78 Vibrionaceae strains. Presence of different Aly and Oal domains is indicated by colored rectangles. Alternating gray and white bars indicate different populations. The left phylogeny depicts the topology (without proper branch lengths) of the maximum likelihood ribosomal protein timed phylogeny on the right.

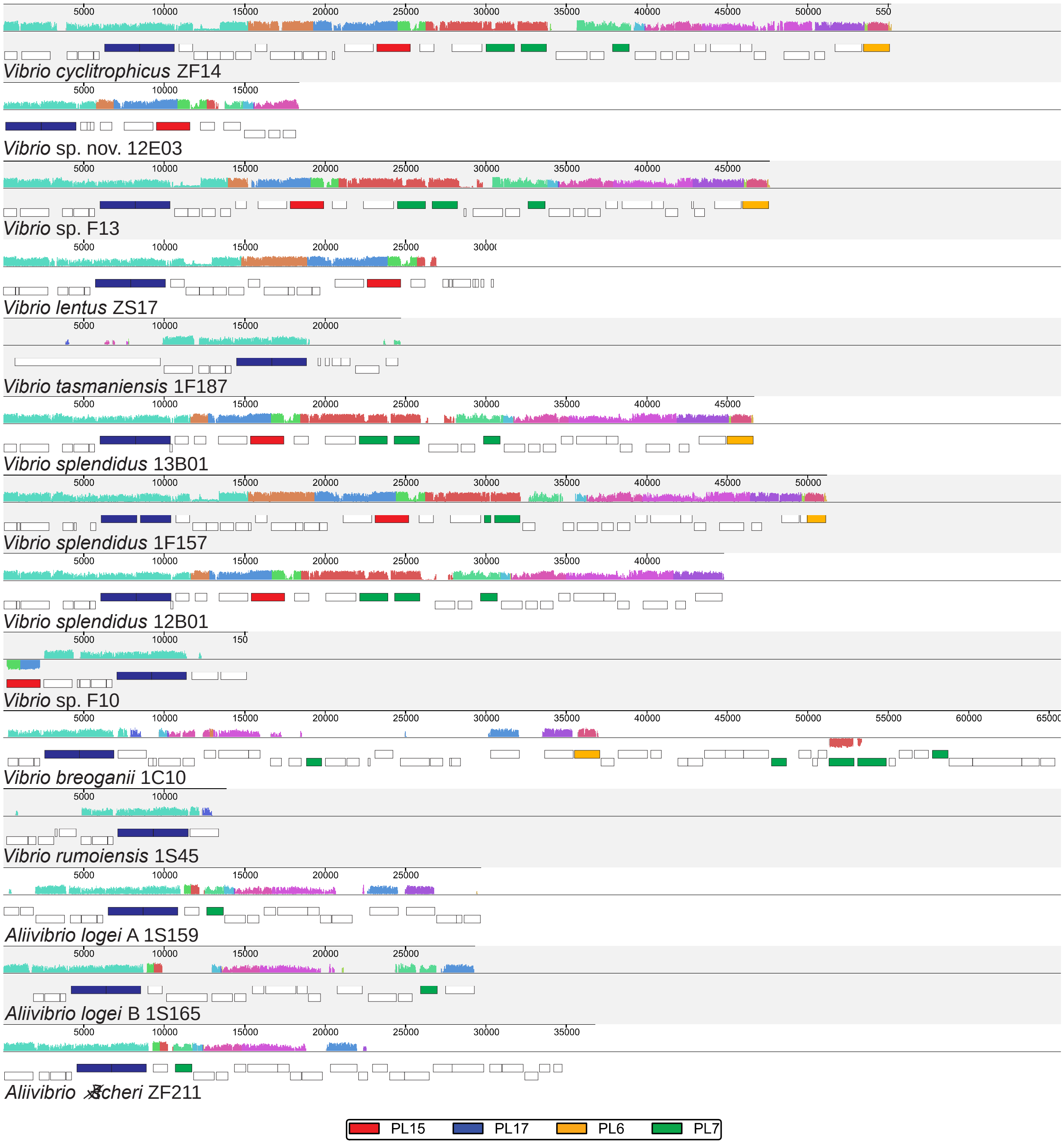

Supplementary Figure 2. Alignment of genetic islands, shared among different vibrio populations, visualizes the core and the flexible parts of enzyme pathways for the catabolism of alginate. We identified contigs with homologs of family PL17 oligoalginate lyases that were previously found to be crucial for alginate catabolism in *Vibrio* strain 12B01[1]. Colored rectangles represent alginate and oligoalginate lyase genes located on the forward (top) or reverse strand (bottom) of the DNA sequence. White rectangles represent additional genes involved in alginate catabolism (e.g. transporters, catabolic enzymes), or genes of unknown

function. The colored bar diagrams, plotted above the genes represent similarity profiles, in that the height of each bar corresponds to the average level of conservation in the corresponding, similarly color coded, genomic sequence[2]. Areas that remain white contain parts that are not shared among the compared sequences. The alignment revealed that some genetic islands contain only the PL17 enzymes, while others encode more elaborate pathway architectures with additional Ola genes of family PL15 or Aly genes of family PL6 or PL7. The presence of PL17 among all populations suggested these enzymes belong to the core part of the alginate degradation pathway. The more variable presence of PL6, Pl7 and PL15 among different populations suggested these enzymes belong to the flexible part of the alginate degradation pathway. It should be noted that genomes of some of the populations (e.g., *V. breoganii*, *V. splendidus*, *V. cyclitrophicus*, and others) contain additional alginate lyase genes that are part of the alginate pathways but which are not shown in this graphic.

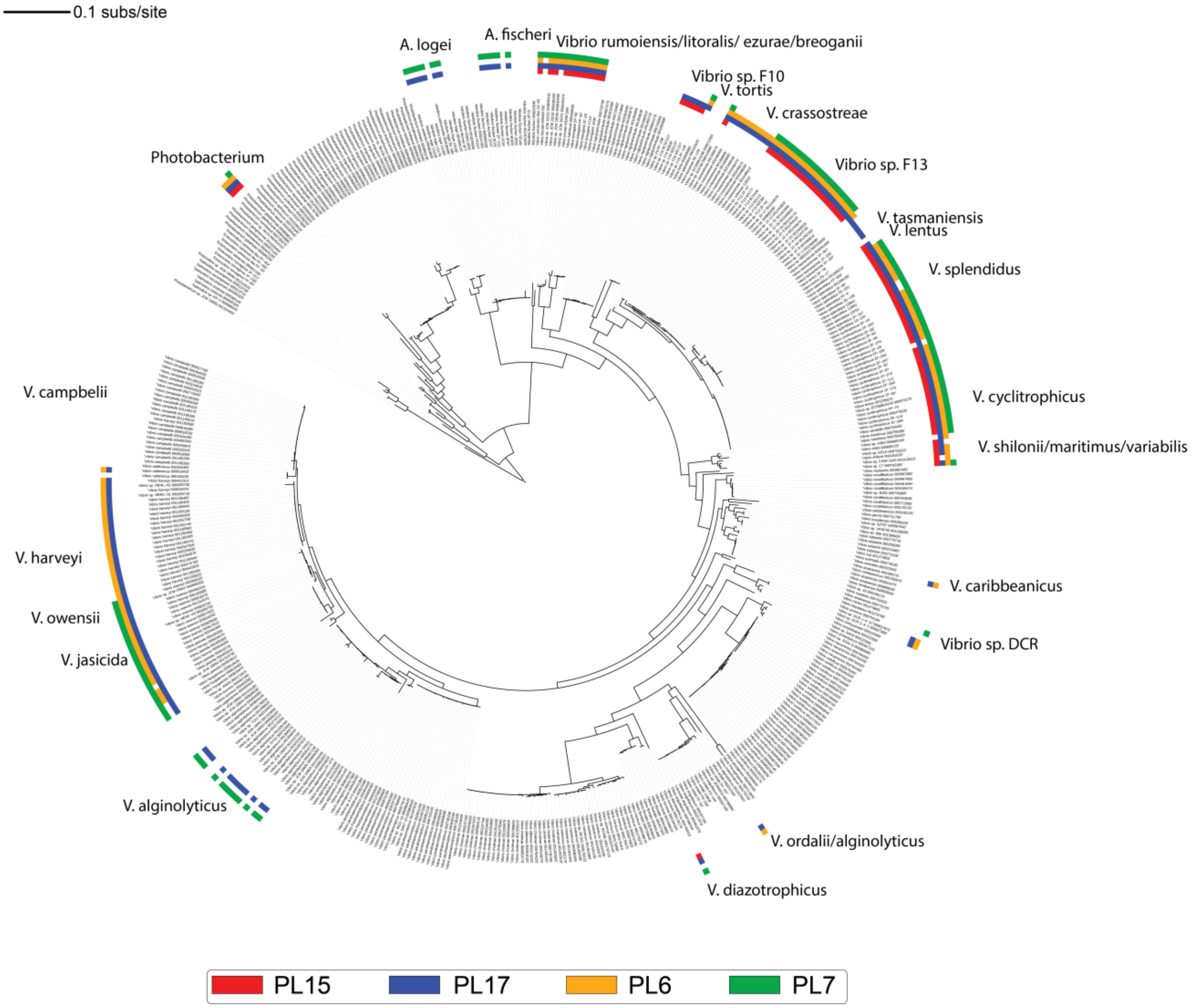

Supplementary Figure 3. Alginate lyase occurrence in Vibrionaceae. Analysis of 446 additional genomes retrieved from Genbank revealed patchy presence of Aly genes in 4 additional, very closely related species, including V. alginolyticus, which, although represented, lacked PL genes in our collection. Because only insufficient physiological and ecological information is available for these isolates, they were omitted from our analysis. Presence of genes indicated by colored rings around phylogeny. Nodes are labeled with species name and Genbank genome identifier or the name of isolate from 84 genomes in the reference phylogeny.

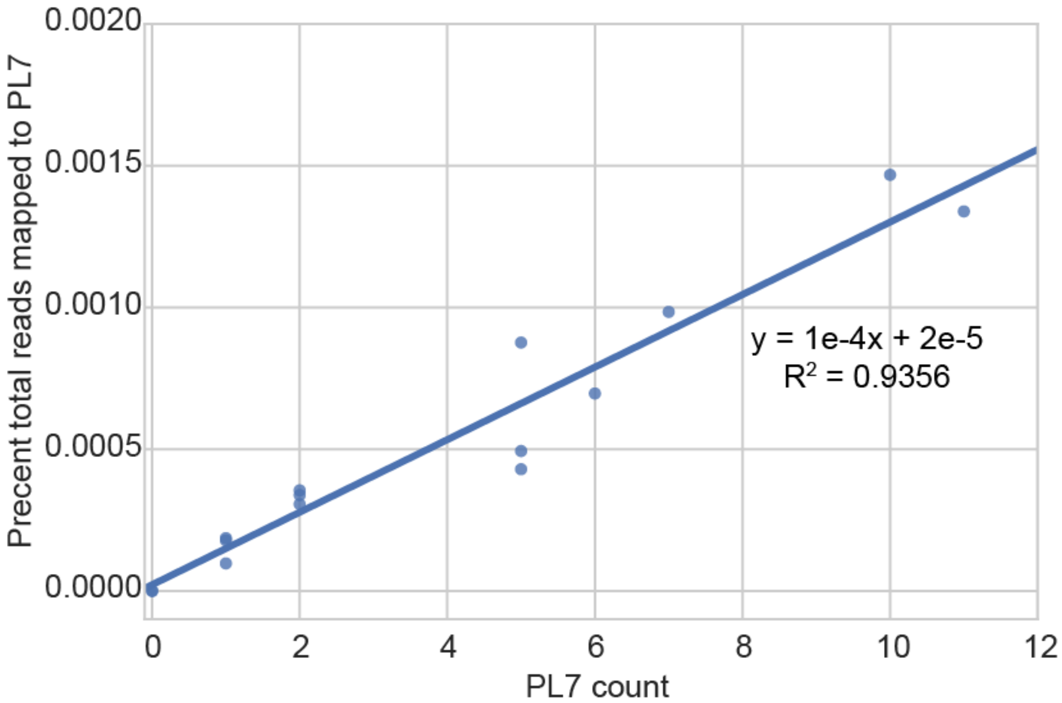

Supplementary Figure 4. Incomplete genomes do not strongly bias copy number estimation of lyase genes. Test of potential bias in alginate lyase copy number estimation due to incomplete genomes. Reads from 15 strains were searched against the complete database of PL7 genes with UBLAST. The number of PL7 domains in each strain was compared to the percentage of reads that mapped to any PL7 in order to ascertain confidence in the inferred PL7 count. Line represents least-square linear regression.

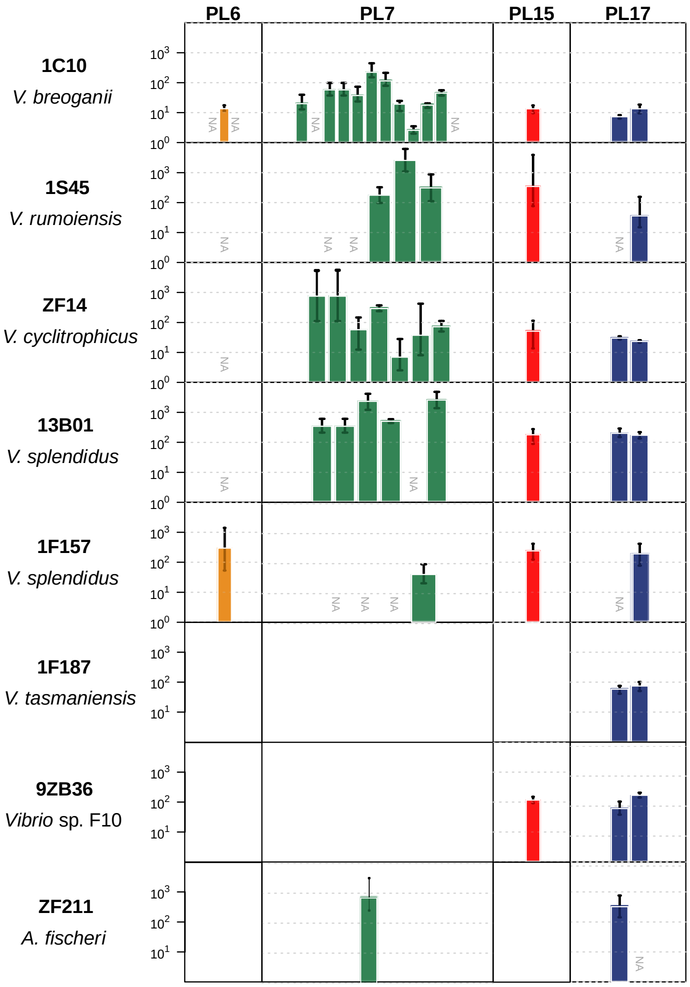

Supplementary Figure 5. Alginate lyase activity in response to alginate oligosaccharides. Expression of alginate lyase and oligoalginate lyase genes measured in response to alginate oligosaccharides as sole carbon source showed induction for all tested Aly and Ola genes. Cells were grown in minimal media with glucose or alginate oligosaccharides. NA = existing protein domain not assayed. No plot = Homologs absent in that strain.

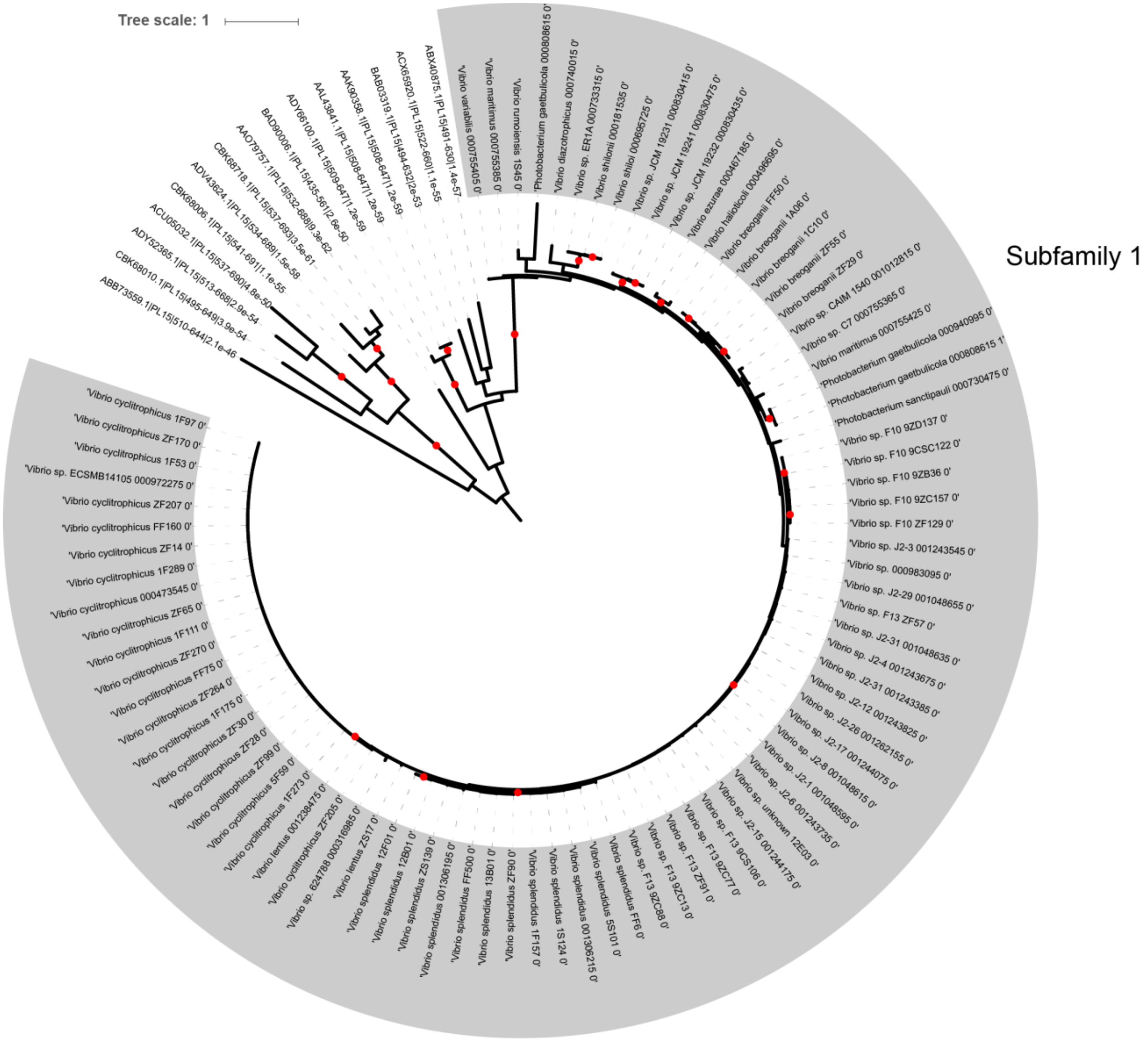

Supplementary Figure 6. Maximum likelihood phylogeny of PL15 Oal domains. Leaves highlighted in grey indicate Vibrionaceae-specific clades (i.e., subfamilies). Red circles indicate bootstrap support > 90.

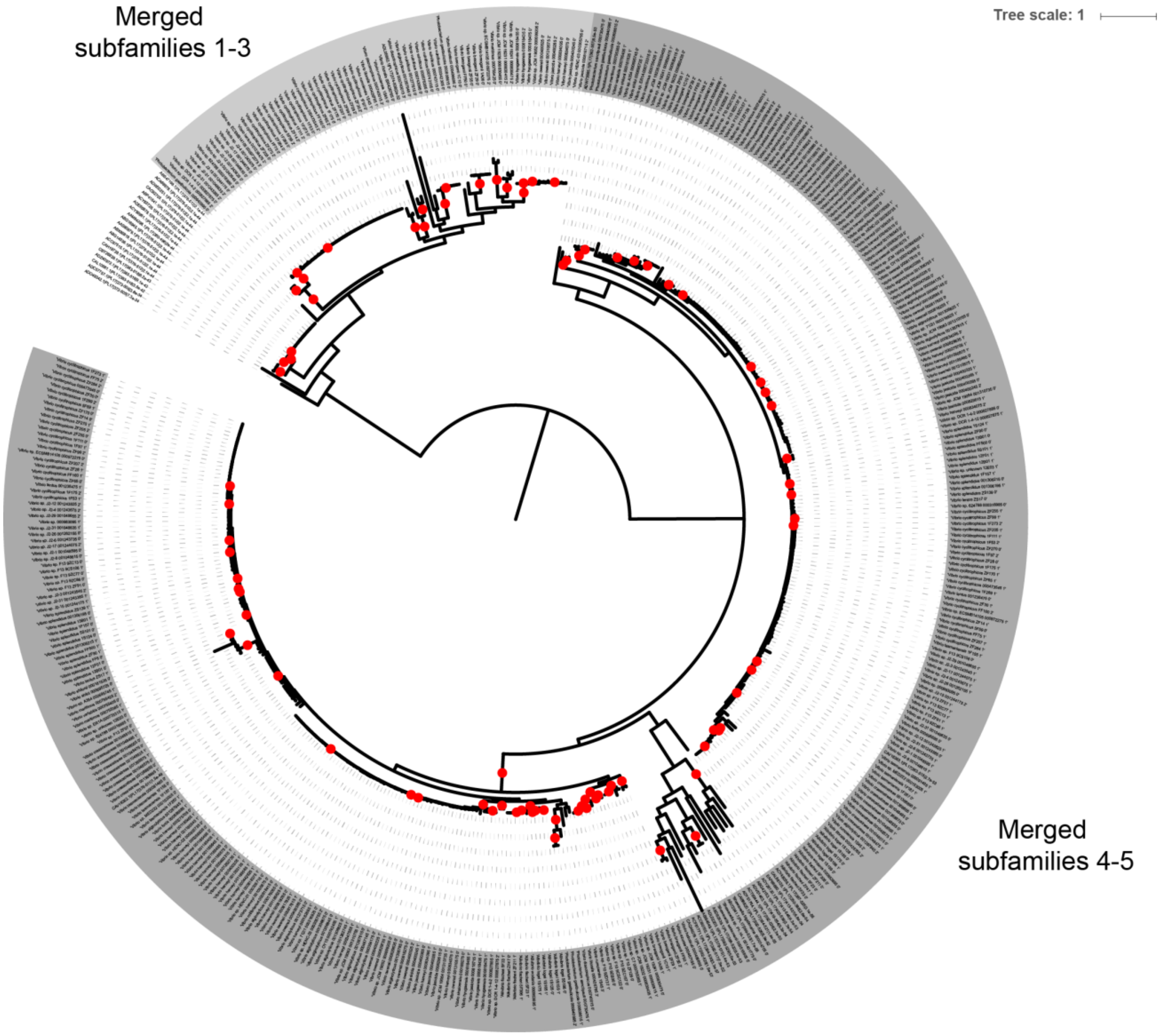

Supplementary Figure 7. Maximum likelihood phylogeny of PL17 Oal domains. Leaves highlighted in grey indicate Vibrionaceae-specific clades (i.e., subfamilies). Red circles indicate bootstrap support > 90.

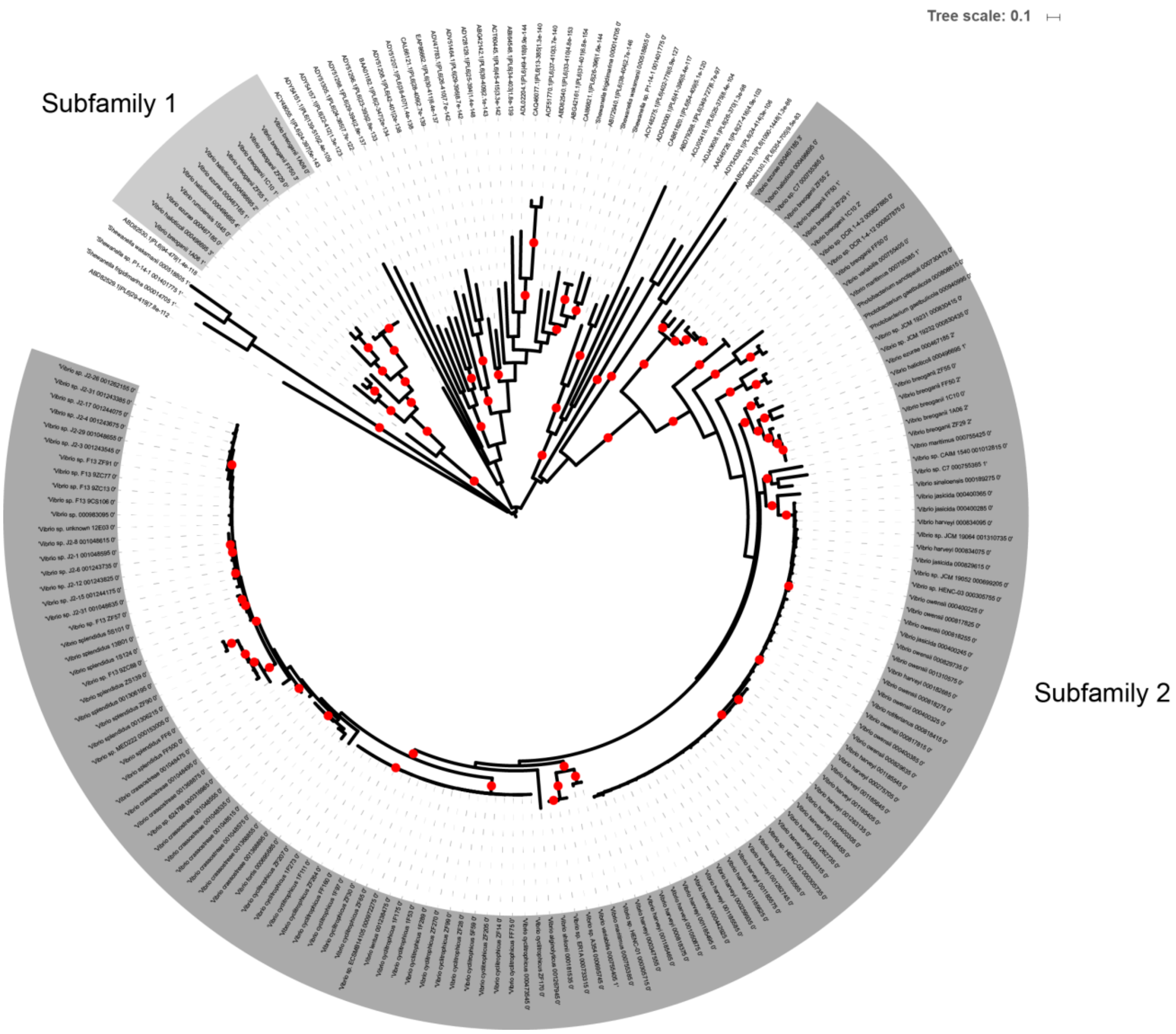

Supplementary Figure 8. Maximum likelihood phylogeny of PL6 Aly domains. Leaves highlighted in grey indicate Vibrionaceae-specific clades (i.e., subfamilies). Red circles indicate bootstrap support > 90.

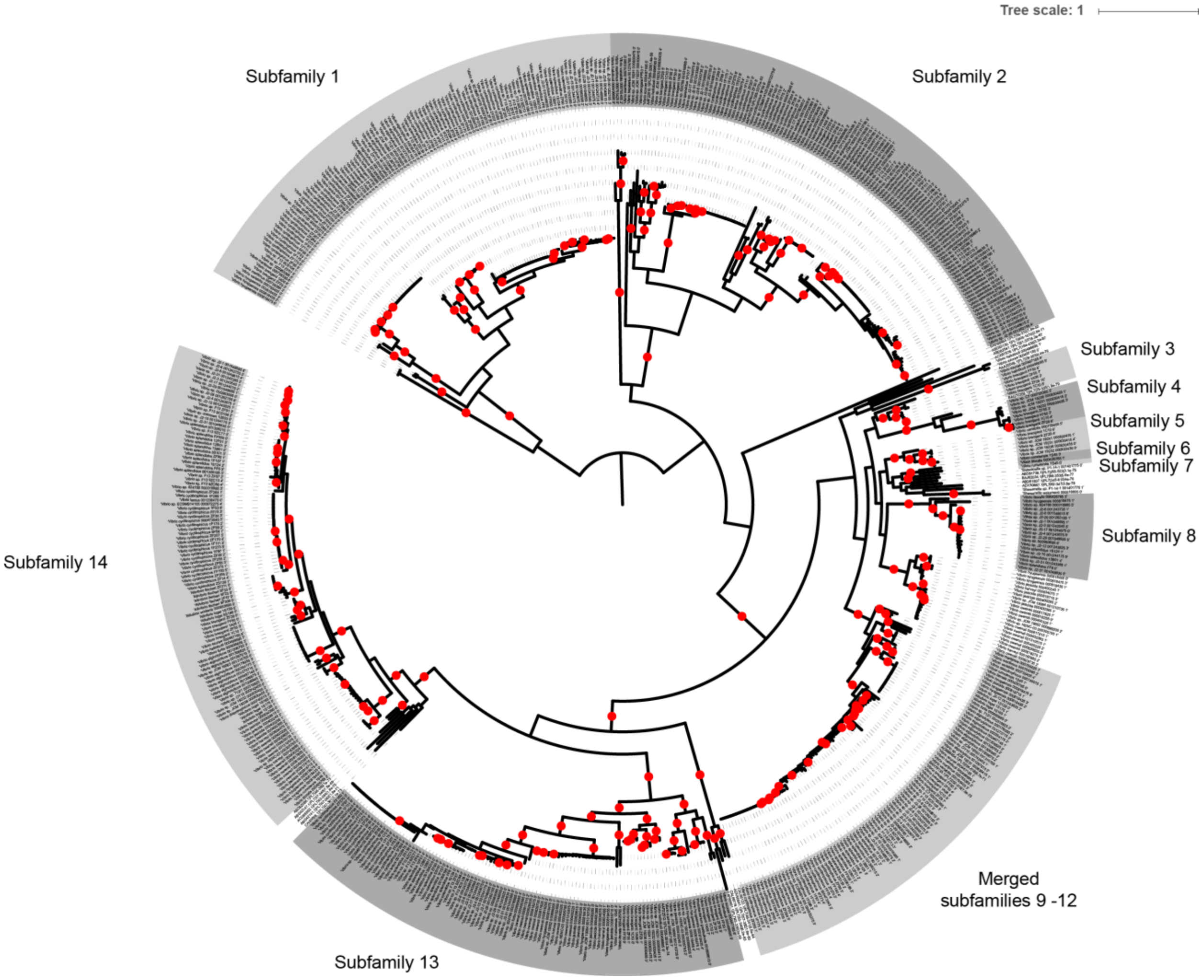

Supplementary Figure 9. Maximum likelihood phylogeny of PL7 Aly domains. Leaves highlighted in grey indicate Vibrionaceae-specific clades (i.e., subfamilies). Red circles indicate bootstrap support > 90.

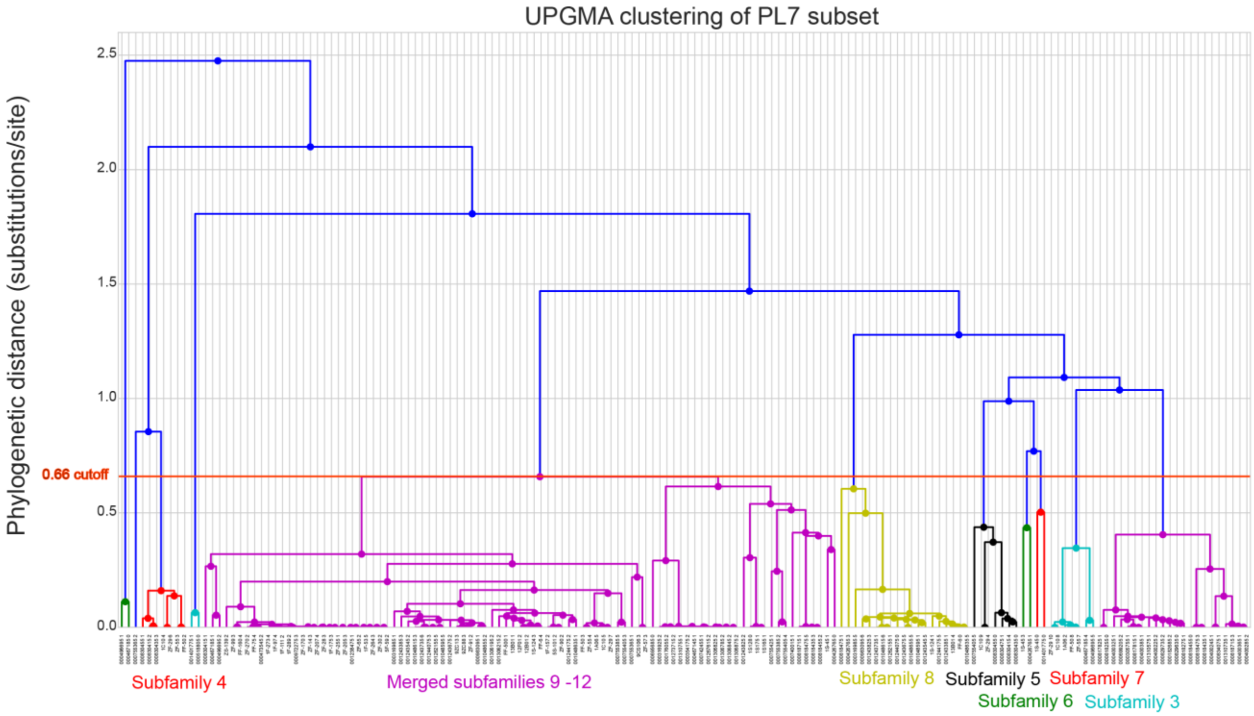

Supplementary Figure 10. UPGMA clustering of PL7 subfamilies 3 – 12. PL7 domains were clustered using phylogenetic distance. Orange horizontal line represents the maximum observed divergence from the root of a separate clade of PL7 inferred to have been born in the ancestor of the *Vibrio* and *Aliivibrio*.

Supplementary Tables

Supplementary Table 1. GC content depletion analysis. A hypergeometric test of GC depletion was carried out on genomic regions from strains 9CS106 and FF50 containing Alys or Oals. Bold rows indicate significant depletion after Bonferroni-correction ($p< 0.05$).

Strain	Chromosome	Start	End	Aly count	Oal count	Region GC content	Chromosome GC Content	Test of GC depletion (Bonferroni-corrected)
**9CS106**	**1**	**1742212**	**1780317**	**3**	**3**	**43.50%**	**44.80%**	**7.55E-07**
9CS106	1	1783747	1795363	1	0	44.20%	44.80%	0.4
9CS106	1	2955232	2966269	1	0	44.00%	44.80%	0.2
9CS106	2	790151	801008	1	0	44%	44.30%	1.19
FF50	1	1613676	1626393	1	0	44.90%	45.50%	1.27
**FF50**	**1**	**2177537**	**2195646**	**1**	**1**	**44.20%**	**45.50%**	**4.00E-03**
FF50	1	2199842	2211062	1	0	44.80%	45.50%	0.95
**FF50**	**2**	**192021**	**202881**	**1**	**0**	**43.00%**	**44.80%**	**1.00E-03**
FF50	2	438021	452318	0	2	45.00%	44.80%	8.9
**FF50**	**2**	**454863**	**465810**	**1**	**0**	**42.00%**	**44.80%**	**2.37E-08**
FF50	2	470497	482068	1	0	43.70%	44.80%	0.127
FF50	2	484045	518229	5	0	44.70%	44.80%	4.75
**FF50**	**2**	**1193193**	**1204761**	**1**	**0**	**43.00%**	**44.80%**	**6.00E-04**
FF50	2	1222411	1233406	1	0	45.40%	44.80%	11.83
FF50	2	1262647	1274725	0	1	44.20%	44.80%	1.59
FF50	ECE1	0	7721	1	0	44.30%	44.30%	6.96
FF50	ECE1	227710	239272	1	0	44.50%	44.30%	8.39

Supplementary Table 2: Genome accession numbers.

Strain	Old Accession	New Accession
12B01	AAMR01000000	N/A
12G01	AAPS01000000	N/A
1F111	AHTI01000000	AHTI02000000
1F53	AICZ01000000	AICZ02000000
1F97	AIDA01000000	AIDA02000000
1F175	AIDB01000000	AIDB02000000
1F273	AIDC01000000	AIDC02000000
1F289	AIDD01000000	AIDD02000000
FF75	AIDE01000000	AIDE02000000
ZF14	AIDH01000000	AIDH02000000
ZF28	AIDI01000000	AIDI02000000
ZF30	AIDJ01000000	AIDJ02000000
ZF65	AIDK01000000	AIDK02000000
ZF99	AIDL01000000	AIDL02000000
ZF170	AIDM01000000	AIDM02000000
ZF207	AIDN01000000	AIDN02000000
ZF205	AIDO01000000	AIDO02000000
ZF264	AIDQ01000000	AIDQ02000000
ZF270	AIDR01000000	AIDR02000000
12F01	AIDS01000000	N/A
FF160	AIDF01000000	AIDF02000000
FF454	AJWN01000000	AJWN02000000
9ZC13	AJYZ01000000	AJYZ02000000

Strain	Old Accession	New Accession
9ZC77	AJZA01000000	AJZA02000000
ZF91	AJZC01000000	AJZC02000000
1.20E+04	AJZD01000000	AJZD02000000
ZF90	AJZF01000000	AJZF02000000
FF238	AJYW01000000	AJYW02000000
5S149	AJYX01000000	AJYX02000000
9CS106	AJYY01000000	CP016228.1 - CP016231.1
9ZC88	AJZB01000000	AJZB02000000
ZS139	AJZE01000000	AJZE02000000
5S101	AJZG01000000	AJZG02000000
FF500	AJZH01000000	AJZH02000000
1S124	AJZL01000000	AJZL02000000
FF6	AJZI01000000	AJZI02000000
1F157	AJZJ01000000	AJZJ02000000
1F267	AJZO01000000	AJZO02000000
1F155	AJZN01000000	AJZN02000000
1F187	AJZM01000000	AJZM02000000
ZS17	AJZQ01000000	AJZQ02000000
1C10	AKXW01000000	AKXW02000000
FF33	AJYD01000000	AJYD02000000

Strain	Old Accession	New Accession
FF162	AJYE01000000	AJYE02000000
FF85	AJYF01000000	AJYF02000000
1F211	AJYG01000000	AJYG02000000
1F230	AJYH01000000	AJYH02000000
5S186	AJYJ01000000	AJYJ02000000
1S45	AJYK01000000	AJYK02000000
ZF55	AJYL01000000	AJYL02000000
ZF29	AJYM01000000	AJYM02000000
9ZD137	AJYO01000000	AJYO02000000
9ZC157	AJYP01000000	AJYP02000000
ZF129	AJYQ01000000	AJYQ02000000
FF167	AJYR01000000	AJYR02000000
FF93	AJYT01000000	AJYT02000000
FS144	AJYU01000000	AJYU02000000
12B09	AJYV01000000	AJYV02000000
FS238	AJYS01000000	AJYS02000000
ZF211	AJYI01000000	AJYI02000000
9ZB36	AJYN01000000	AJYN02000000
FF50	N/A	CP016177.1 - CP016179.1
5F97	N/A	MAJN01000000
5F33	N/A	MAJO01000000
5F306	N/A	MAJP01000000
5F23	N/A	MAJQ01000000
5F146	N/A	MAJR01000000
1S175	N/A	MAJS01000000
1S165	N/A	MAJT01000000
1S159	N/A	MAJU01000000

Strain	Old Accession	New Accession
1S128	N/A	MAJV01000000
FF227	N/A	MAJW01000000
ZF73	N/A	MAJX01000000
ZF47	N/A	MAJY01000000
9CSC122	N/A	MAJZ01000000
5F79	AJZP01000000	MAKA01000000
5F59	N/A	MAKB01000000
FF273	N/A	MAKC01000000
ZF57	N/A	MAKD01000000
ZF255	AIDP01000000	MAKE01000000
13B01	N/A	MAKF01000000
1A06	N/A	MAKG01000000
5F7	N/A	MCGJ01000000
ZF25	N/A	MCGK01000000

*N/A=not applicable

Supplementary Table 3. Oligonucleotides for quantitative amplification of cDNA of alginate lyase genes.

	Forward	Reverse
1C10_PL6_1	TGGCGAAGAGCTTGACCAAT	TACACGGTGAGTCTGGTTGC
1C10_PL6_2	GGTCAGATTGACGGGCTTGA	TAGGTTAGTTGCGCCCACTG
1C10_PL6_4	AAACACGCCACGGTACTCAT	CTGCCGCTCGCTTTTGTATC
1C10_PL7_1	GCTTACTGGGAACACCTGCT	GAGTGCTCGGAAGTGAGCTT
1C10_PL7_2	GTGGGTGACTTGACTGCTGA	TCGCCAAGAGCGATACCATC
1C10_PL7_3	CTCTGAGCTGCGTCAAATGC	GCTTGATAGTGCCCACTGGT
1C10_PL7_4	CGATTACAGCAAGCGTCGTG	ATCTTCTGAACCTTGGCGCA
1C10_PL7_5	GGCACAGCTCAAGCAAACTC	GCCCCAAGCGAACGATTTAC
1C10_PL7_6	TGACGGGCGATCACAAAAGA	TGGCAGCGAGGTGTTAAAGT
1C10_PL7_7	AAACTGGCAGGTAGGTCGTG	GCGTGGTTCATGTGCAAAGT
1C10_PL7_8	ACGCGTCATTGCTAACTCCA	AGTTGCCTTCGCCAACGATA
1C10_PL7_9	TCCGTTCTCCTGTTTCTGGC	TGCCGCGAGTGCTAATAGAG
1C10_PL7_10	CGGCTCGGTTTACTGGAACT	CCCATCTTGCGGGTCTTCTT
1C10_PL15_1	ATTCGGTGGCGAAGGTCAAT	ACTTGTAAGAAGCCGTCGCA
1C10_PL15_2	CGAATGGACCAGAACCACCA	GTGATAACGGATGCGGTTGC
1C10_PL17_1	AAACGGCGTAGTAGGCAACA	GAGACGCATCGTTCATTGCC
1C10_PL17_2	CAAGGTGAGAAAGGCGGTCT	ACCAAACCAGATAAGCGCCA
1C10_PL17_4	CACAACACGCTTGCGGTAAA	GTGACCAATCCACCTCAGCA
12B01_PL7_1	AGGTCAACGGCTCAAACGAT	CAGGTTCTAGTTCAGCCGCA
12B01_PL7_2	TGGGTATTCTCAAGCGCTCC	TCATTCGCATTACCCGTCGT
12B01_PL7_3	AAGCTGGAACCGGTTATGGG	AGCGATATCGGCACGGTTAG
12B01_PL7_4	ACAAGCCATAAGAACGCGGA	CCGACCCATGGATTTGACCA
12B01_PL15_1	GGTCAGTACGGCGAGAACAA	CGCCCGCTTCGATACAAAAG
12B01_PL17_1	AGCTGGTCGCATGTTCTTGA	AAACGACCTGTTGGGTTGGT
12B01_PL17_2	TGCTCAACTGTAGGCGGTTT	CACTTCAGCGAACACACACG
13B01_PL7_1	AGGTCAACGGCTCAAACGAT	CAGGTTCTAGTTCAGCCGCA
13B01_PL7_2	TGGGTATTCTCAAGCGCTCC	TCATTCGCATTACCCGTCGT
13B01_PL7_3	AAGCTGGAACCGGTTATGGG	AGCGATATCGGCACGGTTAG
13B01_PL7_4	CGCGGAAGAGTTTGGCTCTA	CCGACCCATGGATTTGACCA
13B01_PL7_8	AAGGCGCAATCTACTTCGCT	CAATTCCGCCACTTGGGTTG
13B01_PL15_1	TTTCGCAAACGAAGTGGCAG	GGGTCAACTTCGCCAAAACC
13B01_PL17_1	CGTGTGTGTTCGCTGAAGTG	ACGCCGCCTTTGTAGTTGTA
13B01_PL17_2	TCATGGCAACGTCTGTGTGT	AGGCTACCCCAGTTGAATGC
1F157_PL6_1	AGGCAACACGGTTGAGAACA	GCCTTCGATCAAACCATCGC
1F157_PL7_4	TGCAAGCACGCTACGTAAGA	TATGGCTAGCAGGACATGCG
1F157_PL7_5	AGTAACCGGAACGCACGAAT	CCCACTGCAATTTGACCAGC
1F157_PL15_1	CAACCAACTGAAAGGCCGTG	CGTAACCGAAGTCCCACCAA
1F157_PL17_1	GTTACGCGATTCGTCCAACG	ACGCCGCCTTTGTAGTTGTA
1F157_PL17_2	GCATTCAACTGGGGTAGCCT	AACCAGATCAACGCCATCGT
common_GyrB	GGGCGTAACCGTAAGAACCA	ACCACAACCAAGTGCTGTGA

	Forward	Reverse
common_RpoD	AGACACCAATCGGTGACGAC	CTTGTTGCCGTTGCAGAGTC
1F187_PL17_1	AAATTCGGCGGTCGTTACCT	AAGCCGTGTACTGAGTCTGC
1F187_PL17_2	TACATGGCGGAAGTTGGCAT	GGAACAAACGACCCGCTAGA
ZF14_PL6_1	ATGGGAATACGTTGGCCGTT	ACGACTTCGTCGCCATCTTT
ZF14_PL7_1	GGTACGGCACTACGACACAA	CCGGTTGTGCATTGCTTTGA
ZF14_PL7_2	ACCGGTAATGCGAACGAAGT	CGTGTGCAAAGTAAACCGCA
ZF14_PL7_3	CAGAGCGTCACGATACGGTT	TCTTCAGCATAGCCCGTTGG
ZF14_PL7_4	CTTGACCCTGCAAAAGCACC	TCGGGTGAACGTAAGGTGTG
ZF14_PL7_5	AAGCCGGTACGTGTTGTTCT	GGCTGACAGTCGCTACACAT
ZF14_PL7_6	TGAAGGGCATTTCTGGGCAA	CTGTTGGTTTGCCTTCTGGC
ZF14_PL15_1	TCAAGCACTATGCGGGTGTT	CGTAACCGAAGTCCCACCAA
ZF14_PL17_1	AGCGAGTGGTGAAGTGGATG	TAGCACCAGTGCGAGTGAAG
ZF14_PL17_2	ACGATCTTCCCGTTCACCAC	CTTTGCCTGAAGCCACGTTC
ZF14_PL17_4	GGGATAGAGACCGACCCACT	GGTTCCCCAGTAAGCACCTC
ZF14_GyrB	GGTTCTTCACGCTGGTGGTA	CCGTGGCTGTAGGTTTGAGT
ZF14_RpoD	TGGAACAGTTTGACCGCGTA	CATCGTCGTCTGGGTCAACA
1S45_PL6_1	TCATGGGCGGGCAATATACC	TCATGAGTGCCAAAGCGAGT
1S45_PL7_2	GCAAGTGGTGATGGGCAATG	ACCCAATAACGCCACCTGAG
1S45_PL7_3	GTGTGGGGCAATACATGGGA	TTGTCATGCTTAGCGGTCGT
1S45_PL7_4	TGATGATAAGCCGGGTCGTG	AACGCGGAAAACCATTGCTC
1S45_PL7_8A	GATGGCTGGAACGATAGCGA	GATTCCAGAGCCAGTCACCC
1S45_PL7_8B	CAATGGCCGCGTAGCTTTAC	GAACTCGATGAGCCACCACA
1S45_PL15_1	GAGAACGATACCTCGGCAGG	AATAAGAAGACGTGGGCGGG
1S45_PL17_1	TACAAGTGGCAGTGAGCCAG	CTTGGCCAGAAAGGCAAACC
1S45_PL17_2	TATGCCACGGTGTACCCAAC	CGGAGGGTTAGTATCACGGC
1S45_PL17_3	AACCAGTGCGACTATTGGGG	AGTTTACCCCCTCAGCAAGC
1S45_PL17_4	CCGCCGTTGGAATTATTGGC	AGCAGTGATTGTAGGGCGTC
1S45_GyrB	GCGATTCTGCCGCTTAAAGG	CACGTCCGATACCACAACCA
1S45_RpoD	GTATGCGTTTCGGCATCGAC	TACTCGTCAAGGAAGCTGCG
ZF211_PL7_3	GATCCACGCGAAGAAACACG	CCCCAAACAGGGTAAGCGAT
Zf211_PL15_1	AGGCAAGGAACAAGGCTACC	TGTCATCAGCATCAGGAGCG
ZF211_PL17_1	ATTGGCGGTTTACGCAGAGA	GAAACAAACGACCCGTTGGG
ZF211_PL17_2	GCTCGTGGGTTGGTAAAAGC	GTTGTGCGTCTTCAGCTTGG
ZF211_GyrB	GGGTGAGATGAACCCTGAGC	CGCGTAATGCGTTCTCTTCG
ZF211_RpoD	AGGCAAGGAACAAGGCTACC	TGTCATCAGCATCAGGAGCG
9ZB36_PL15_1	ACAACAAGATGACCGAGCGT	TGACGCTAACGTTTCACCGA
9ZB36_PL17_1	GCATCAAGGGTGAGGGTGAA	GATGCTGATAGCCAGCGTCT
9ZB36_PL17_2	ACCGTTACGAGCAAGCTGAA	TAGGCTGCCCCAGTTGAATG
9ZB36_GyrB	AGCAAAACGACTGCGTGAAC	TGCTTGAATACCGCCTTCGT
9ZB36_RpoD	ATGCGTGAAATGGGTACCGT	TCAGCAACTGCGCTTTGAAC

Supplementary References

1. Wargacki AJ, *et al.* An engineered microbial platform for direct biofulel production from brown macroalgae. *Science* **335**, 308-313 (2012).

2. Darling AE, Mau B, Perna NT. progressiveMauve: multiple genome alignment with gene gain, loss and rearrangement. *PLoS One* **5**, e11147 (2010).
